# Biomaterials for in situ cell therapy

**DOI:** 10.1002/bmm2.12039

**Published:** 2023-07-19

**Authors:** Chang Wang, Siyu Wang, Diana D. Kang, Yizhou Dong

**Affiliations:** 1 Department of Oncological Sciences, Icahn Genomics Institute, Precision Immunology Institute, Tisch Cancer Institute, Friedman Brain Institute, Icahn School of Medicine at Mount Sinai, New York, New York, USA; 2 Division of Pharmaceutics & Pharmacology, College of Pharmacy, The Ohio State University, Columbus, Ohio, USA

**Keywords:** biomaterial, cell therapy, drug delivery, in situ

## Abstract

Cell therapy has revolutionized the treatment of various diseases, such as cancers, genetic disorders, and autoimmune diseases. Currently, most cell therapy products rely on ex vivo cell engineering, which requires sophisticated manufacturing processes and poses safety concerns. The implementation of in situ cell therapy holds the potential to overcome the current limitations of cell therapy and provides a broad range of applications and clinical feasibility in the future. A variety of biomaterials have been developed to improve the function and target delivery to specific cell types due to their excellent biocompatibility, tunable properties, and other functionalities, which provide a reliable method to achieve in vivo modulation of cell reprogramming. In this article, we summarize recent advances in biomaterials for in situ cell therapy including T cells, macrophages, dendritic cells, and stem cells reprogramming leveraging lipid nanoparticles, polymers, inorganic materials, and other biomaterials. Finally, we discuss the current challenges and future perspectives of biomaterials for in situ cell therapy.

## 1 | INTRODUCTION

Cell therapy has been applied in the clinic to treat diverse diseases.^[1–6]^ Since 2010, several cell therapies based on T cells, hematopoietic stem cells, progenitor cells, and fibroblasts have been approved by the FDA for cancer immunotherapy, bone marrow transplantation, or other specific diseases.^[3,7–10]^ Although the efficacy of adoptive cell therapy has been supported by numerous preclinical and clinical data with impressive therapeutic outcomes,^[11]^ current approaches require complicated protocols to engineer cells ex vivo to maintain cell viability and functions, which may induce severe adverse effects. This limits the broader application of cell therapy and the number of potentially benefiting patients.^[12]^ To address these limitations, there is an increasing interest in developing in situ cell therapy.^[13]^

Compared to adoptive cell therapy, in situ cell therapy possesses multiple advantages.^[14]^ First, in situ cell therapy simplifies the complex process of cell engineering by eliminating the need for cell isolation, modification, and amplification in vitro.^[15]^ Second, it improves outcomes and accelerates therapeutic efficiency by decreasing the time for the amplification of transfected specific cells. Third, it reduces therapeutic resistance, which can provide a simpler and faster approach to generating specific cellular therapeutics that match the individual patient’s requirements against the diversity of tumor phenotypes.^[16]^ In addition, in situ cell therapy can minimize invasive damage since major surgery is not required.^[17]^ Finally, it raises the potential to improve the long-term well-being of patients.^[18]^

**FIGURE 1:**
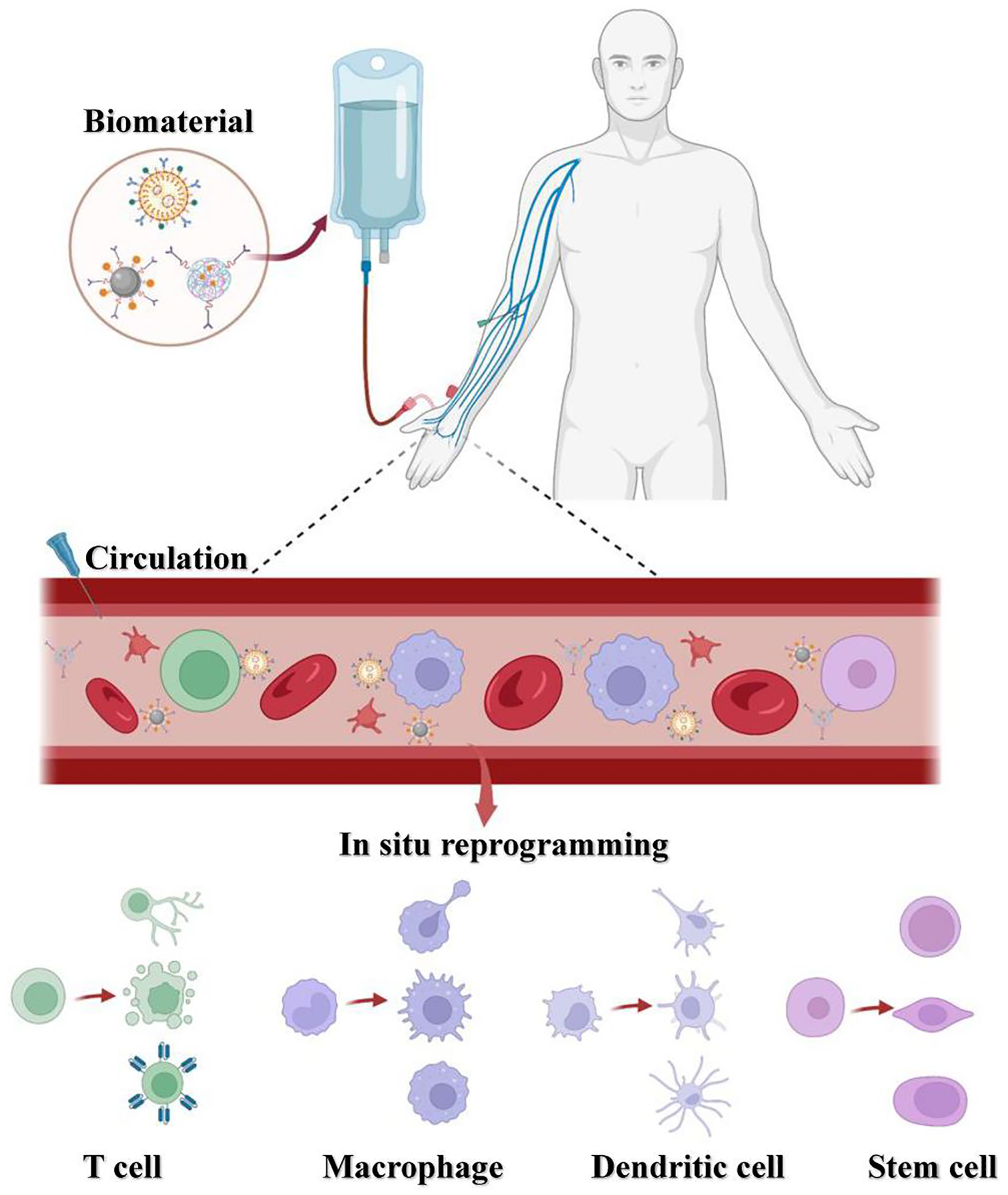
Schematic diagram illustrating the reprogramming of T cells, macrophages, dendritic cells, and stem cells in situ using agents carried by biomaterials. Biomaterials with certain physicochemical properties allow them to target specific cells. Also, they can be modified with diverse ligands for improved cell specificity. Upon the administration of the biomaterials into the patient, their targeting ability facilitates binding to the specific cells, resulting in the subsequent release of encapsulated cargo such as small molecules, nucleic acids, or other components. This release induces in situ cell reprogramming, leading to the formation of multifunctional cells, and ultimately enabling effective in vivo therapy.

To achieve in situ cell therapy, researchers have explored numerous biomaterial-based platforms to modulate cell functions in vivo.^[19]^ Biomaterials, including lipid nanoparticles (LNP), polymer nanoparticles, and inorganic nanoparticles exhibit unique properties such as biocompatibility, biodegradability, and tissue and cell specificity. Particularly, physical and chemical characteristics such as morphology and functional composition of biomaterials could be tuned by controlling material chemistry, particle size, or surface charge.^[19]^ Therefore, biomaterials may enhance the specific targeting of certain cells with diverse ligand modifications and enable the delivery of small molecule agents, nucleic acids, and proteins into specific cells in vivo, ultimately realizing in situ cell therapy (Figure 1).^[20–22]^ These inherent advantages facilitate the effective utilization of these biomaterials for the targeted delivery of therapeutics to diverse cell types such as T cells, macrophages, dendritic cells (DC), and stem cells. Consequently, significant advances have been made in the fields of cancer immunotherapy, bacterial therapies, and anti-inflammatory treatments.^[14,23]^

Here, we provide an overview of recent advances in biomaterials used for in situ cell reprogramming. Specifically, we focus on biomaterials to engineer four specific cell types: T cells, macrophages, DC, and stem cells. Additionally, we describe the current challenges of this field and future directions for the applications of biomaterials for in situ cell therapy.

## 2 | T CELL BASED THERAPY

T cell-based therapy has been approved by the FDA to treat acute lymphoblastic leukemia, lymphoma, and multiple myeloma.^[24,25]^ T cell engineering has opened up new possibilities for personalized medicine, where a patient’s T cell can be genetically modified to treat specific diseases.^[26]^ For example, chimeric antigen receptor (CAR) T cell therapy, tumor-infiltrating lymphocyte (TIL) therapy, and T cell receptor (TCR) therapy are some strategies to activate the T cells.^[27,28]^ However, the current successes of T cell therapy depend on ex vivo engineering such as lentiviral vector mediated gene modulation,^[29,30]^ which raises considerable concerns regarding the safety, efficacy, and costs associated with T cell therapy.^[31]^ Biomaterials provide the opportunity to address this challenge by targeted delivery of therapeutics (small molecule agents, siRNA, plasmid DNA, mRNA, etc.) to T cells in vivo.^[32,33]^ In this section, we highlight different biomaterials utilized for the delivery of therapeutics to T cells in situ.

LNPs show great potential as a biomaterial for the delivery of nucleic acids to T cells in situ. In 2010, Kim et al. developed a method using LFA-1 integrin-targeted immunoliposome to deliver anti-CCR5 siRNA, resulting in selective targeting of T cells in vivo.^[34]^ This approach showed long-lasting T cell specific gene silencing effects and improved resistance to the humanized mouse model of HIV infection. Thereafter, multiple ionizable lipids formulated LNPs were conjugated with targeting ligands such as anti-CD4 and anti-CD3 antibodies for in situ modulation of T cell function through the delivery of nucleic acids such as siRNA and mRNA.^[35,36]^ For example, in 2019, Munakata et al. developed an LNP formulation containing D35 (D35LNP) and Type-A CpG oligodeoxynucleotides (ODN). The results showed that D35LNP activated T cells in vivo for anti-tumor treatment in a CD8 T cell-dependent manner.^[37]^ In 2020, Billingsley et al. reported ionizable lipids (C14–4) as a component of LNP for CAR mRNA delivery to primary human T cells.^[38]^ In 2022, Rurik et al. constructed T cell-targeted LNPs, which were surface-modified with anti-CD5 antibodies and loaded with mRNA encoding fibroblast activation protein (FAP)-CAR. Following intravenous administration, these LNPs delivered FAP-CAR to 17.5%–24.7% of splenic T cells in mice. In a mouse model of cardiac fibrosis, cardiac function was restored and fibrosis was reduced after 2 weeks of LNP administration.^[39]^ Additionally, in 2019, Lokugamage et al. delivered sgRNA and siRNA to T cells using LNPs in the absence of targeting ligands.^[40]^ They synthesized 16 ionizable lipids bearing different amino heads and mixed them with other lipids in various ratios to produce 104 distinct LNPs. By encapsulating sgRNA against Green Fluorescent Protein (GFP) and DNA barcodes, these LNPs were high throughput screened in vivo. It was found that adamantane-containing LNP showed high delivery efficiency in splenic T cells. Recently, Li et al constructed phospholipid-derived biomimetic nanoparticles (PL1), which effectively delivered CD137 or OX40 mRNA to the tumor-infiltrating T cell. The administration of PL1-OX40 mRNA with anti-OX40 agonist antibodies activated T cells in vivo, enhancing T cell-mediated cancer immunotherapy.^[41]^

**FIGURE 2:**
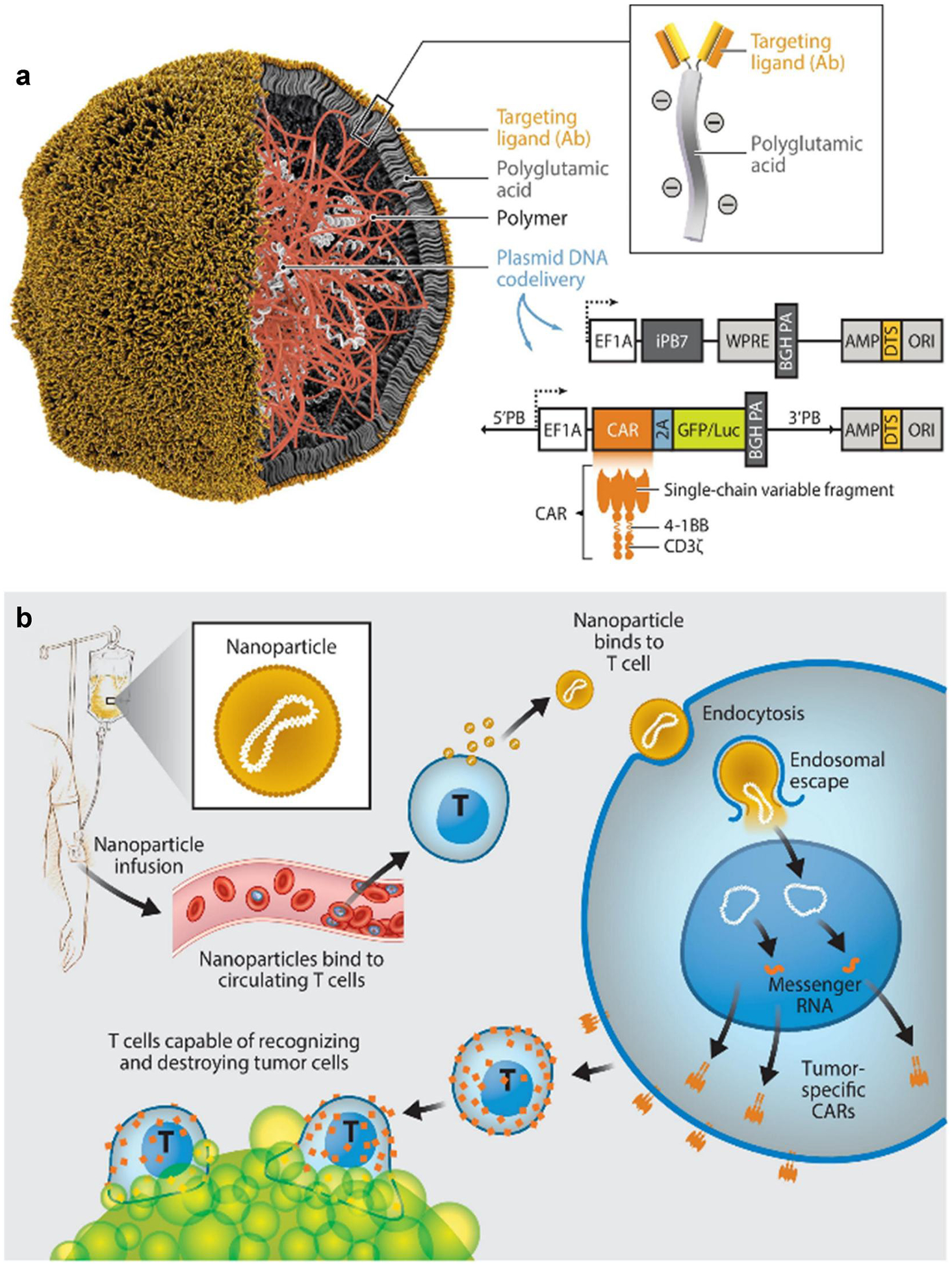
(a) Construction of the T-cell-targeted DNA-carrying polymer nanoparticles. (b) Reprogramming of T cells in situ to express tumor-specific CARs using DNA-carrying polymer nanoparticles. (Reproduced with permission from ref 15, 45.^[15,45]^ Copyright © 2021 by Annual Reviews. Distributed under a Creative Commons Attribution License 4.0 (CC BY) https://creativecommons.org/licenses/by/4.0/).

Polymer-based biomaterials have also been investigated for T cell therapy in vivo for decades. Researchers have used various polymers including polyethyleneimine (PEI), poly(lactic-co-glycolic acid) (PLGA), L-phenylalanine polymer, and natural polymers like chitosan, alginate, and hyaluronan for in vivo T cell therapy.^[42,43]^ In 2011, Zhou et al. developed poly (amidoamine) (PAMAM) dendrimers as a delivery system for anti-virus siRNA to silence CD4, which prevented the consumption of CD4^+^ T cells in vivo.^[44]^ In 2017, Smith et al. developed poly (β-amino ester) (PBAE) nanoparticles coated with anti-CD3e F(ab’)2 fragments for selectively targeting T-cell populations.^[45]^ These nanoparticles delivered plasmids encoding a 194–1BBz CAR and a piggyBac transposase to T cells (Figure 2a). Intravenous injection of the nanoparticles showed comparable anti-tumor effects to ex vivo CAR T cell in a mouse model of B cell lymphoblastic leukemia (Figure 2b). Xie et al. modified PEI with the transferrin receptor ligand (TfR ligand) for siRNA delivery in activated T cells (ATCs).^[46]^ This system enhanced endosomal escape, cytoplasmic delivery, and siRNA encapsulation efficiency in ATCs, which resulted in efficient gene knockdown in primary human ATCs. In 2020, Parayath et al. constructed PBAE nanoparticles decorated with anti-CD8 antibodies for in situ T-cell programming. These nanoparticles encapsulating CAR or TCR mRNA showed targeted delivery and activation of T cells.^[47]^ In mouse models of human leukemia, this in vivo T-cell programming approach displayed similar levels of disease regression compared to the adoptive transfer of ex vivo engineered T cells.

Inorganic biomaterials provide unique characteristics, including a high surface space to volume proportion and high avidity display, which make them advantageous for in vivo T cell therapy.^[48]^ Among these inorganic biomaterials, gold nanoparticles are particularly favored due to their small size, inertness, and nontoxicity. They can be bound to a diverse category of cargo, such as proteins, peptides, nucleic acids, and small molecule agents.^[49]^ For instance, Yang et al. developed gold nanoparticles (amph-NPs) coated with amphiphilic organic ligands, enabling efficient transport of small molecule agents to target T cell populations.^[50]^ The uptake of amph-NPs by CD8^+^ T cells increased 40-fold compared to unmodified nanoparticles, resulting in enhanced polyfunctionality of cytokines in a cancer vaccine model.

Various biomaterials have been modified for in vivo targeting or functional modulation of T cells by ligand modification. In situ T cell therapy by biomaterials, which utilizes the patient’s endogenous immune system, has a great prospect to provide targeted and effective treatments. Moreover, it can be combined with other existing treatments such as chemotherapy or radiotherapy to improve overall therapeutic efficacy. However, challenges still exist, including the identification of specific surface markers of T cell subpopulations, the design of optimal physicochemical properties of biomaterials to promote interactions with T cells in vivo, maximizing the delivery in T cells and minimizing uptake by other cells.

## 3 | MACROPHAGE BASED CELL THERAPY

Macrophages have been identified as attractive targets for treating various diseases such as cancer, inflammation, and infectious diseases.^[51–53]^ Recently, a CAR macrophage-based clinical trial for solid tumors has been initiated for macrophage-based cell therapy.^[54]^ Macrophages, as an essential element of the innate immune system, can be generally classified as M1 type (pro-inflammatory) and M2 type (anti-inflammatory).^[55]^ Due to their unique phenotypes in different diseases, macrophages are promising candidates for in situ cell reprogramming.^[56]^ For example, reprogramming tumor-associated macrophages from M2-type macrophages to M1-type macrophages could enhance the immune system’s ability to inhibit tumor growth.^[57]^ To achieve in situ macrophage therapy, researchers have developed various biomaterials for the targeted delivery of cytokines, small molecules, or nucleic acids to enable macrophage reprogramming at the site of disease.^[58–60]^

In 2010, mannosylated ligand-modified cationic solid lipid nanoparticles (SLN) were found to improve active targeting DNA delivery to pulmonary macrophages in vivo.^[61]^ Later on, Whitehead et al. developed several types of LNPs that were able to induce high efficiency of gene silencing in various cell subtypes in mice, including macrophages.^[62]^ In 2018, Kedmi et al. reported a targeting approach through non-covalent interactions between LNP and targeting antibodies. They coated D-Lin-MC3-DMA (MC3) LNPs with a recombinant protein (named ASSET) that can bind to the Fc domain of an antibody. By tuning specific antibodies, the modified MC3 LNPs were able to target various cell types.^[63]^ Using this approach, Veiga et al. showed the targeted delivery of FLuc and IL-10 mRNA specifically to leukocytes (Ly6c+) by LNPs. The LNPs were formulated with MC3 and coated with anti-Ly6c^+^ monoclonal antibodies.^[64]^ In a colitis model induced by dextran sodium sulfate, these LNPs effectively induced the expression of the anti-inflammatory cytokine IL-10 specifically in Ly6c^+^ inflammatory leukocytes, which enabled macrophage polarization and symptom alleviation. A separate study by Zhou et al. reported a mannose-modified MC3 LNP encapsulating HMGB1-siRNA, which targeted liver macrophages via mannose receptors. In a non-alcoholic steatohepatitis model, the formulated LNP silenced HMGB1 protein expression and improved liver function.^[65]^ In addition to MC3, other lipids can be utilized for in vivo macrophage cell therapy. In 2018, Luo et al. utilized a cationic lipid-PEG-PLGA nanoparticle-based system to deliver DNA plasmids for gene editing. This system applied a macrophage-specific promoter to specifically express Cas9 and a guide RNA (sgNtn1) against Ntn1 in macrophages in vivo.^[66]^ After intravenous injection, only Ntn1 in monocytes and macrophages was knocked down. In another study, Xu et al. constructed cationic LNP to deliver CRISPR–Cas9 mRNA and gRNA to downregulate NOD-, LRR-, and pyrin domain-containing 3 (NLRP3) expression in macrophages in vivo.^[67]^ After intravenous administration of LNPs, the disruption of the NLPR3 gene in macrophages resulted in the alleviation of acute inflammation associated with lipopolysaccharide (LPS)-induced septic shock and monosodium urate crystal (MSU)-induced peritonitis. Moreover, it improved insulin sensitivity and reduced adipose tissue inflammation in high-fat diet (HFD)-induced type 2 diabetes (T2D).

**FIGURE 3:**
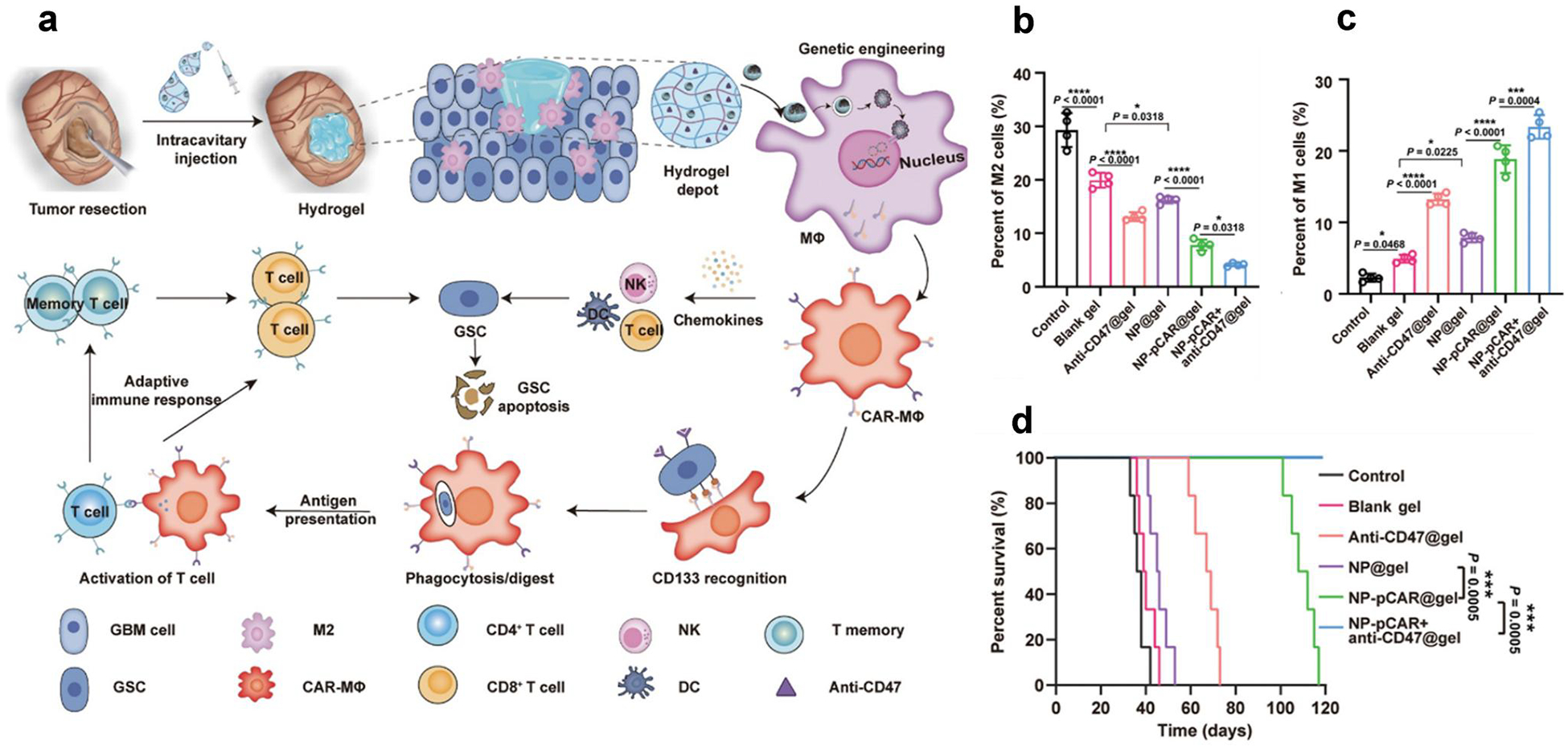
(a) Illustration of the CD133-specific CAR-Ms by hydrogel-nanoporter, (b) M2-like macrophages and (c) M1-like macrophages in tumor sites, (d) Survival rate of mice after treatments (Reproduced with permission from ref 75.^[75]^ Copyright © 2022, The American Association for the Advancement of Science).

Various polymers have also been studied to induce macrophage polarization for disease treatments.^[68]^ In 2015, Jain et al. developed tuftsin peptide-conjugated alginate nanoparticles with plasmid DNA encoding IL-10 for autoimmune diseases.^[59]^ After the systematic administration, the nanoparticles were observed to accumulate in the inflamed joints of arthritic rats. These nanoparticles reprogrammed macrophages from M1-like phenotype to M2-like phenotype, leading to the downregulation of inflammatory cytokines and overall protection of the joint from inflammation. In 2018, Tran et al. used hyaluronic acid- PEI nanoparticles carried with plasmid DNA encoding IL-4 to inhibit inflammation. These polymeric nanoparticles could target macrophages due to the high affinity of HA and CD44, which is overexpressed on macrophages.^[69]^ In 2019, Zhang et al. reported PBAE-based nanocarriers that specifically target M2 tumor-associated macrophages.^[58]^ This nanocarrier loaded with mRNAs encoding M1-polarizing transcription factors of interferon regulatory factor 5 and inhibitor kappa B kinase *β* (IKKβ), reprogrammed immunosuppressive M2 phenotype to the antitumor M1 phenotype. In a glioblastoma mouse model, the combination of mRNA nanocarriers and radiotherapy enabled the genetic programming of macrophages, resulting in inhibited tumor growth and more than double the survival rate of mice compared to radiotherapy alone. In 2023, Zhang et al developed an approach using PBAE-based nanoparticles for in situ reprogramming of M2 macrophages.^[70]^ In this study, they employed a plasmid encoding dCas9-KRAB-sgPI3Kγ (sgPI3Kγ, sgRNA targeting PI3Kγ), which was complexed with a nuclear localization signal (NLS) conjugated PBAE. These complexes were surface modified with a peptide (CRV) capable of precise macrophage targeting through its interaction with retinoid X receptor beta. After intravenous administration, the complex targeted M2-like macrophages and led to the deactivation of PI3Kγ and the induction of macrophage polarization. In a Lewis Lung Cancer (LLC) tumor mouse model, flow cytometry analysis revealed that the tumor microenvironment was remodeled, which shifted the anti-inflammatory microenvironment to an inflammatory state. Besides, polymer-based hydrogels and microneedles have also been studied for in vivo macrophage therapy.^[71]^ For instance, Jin et al synthesized a cationic polypeptide-based melittin-RADA32-doxorubicin (DOX) hydrogel (MRD hydrogel), which depleted M2 macrophages in vitro and in vivo.^[72]^ Lately, Gao et al. developed an injectable temperature-responsive chitosan polymer hydrogel, which is loaded with lipid-immune regulatory factor 5 (IRF5) mRNA and C–C chemokine ligand 5 (CCL5) siRNA (LPR) nanoparticles.^[73]^ This hydrogel system allowed controlled release of nanoparticles. These released nanoparticles upregulated IRF5 expression and downregulated CCL5 expression in tumor tissues, which led to an increase in M1 phenotype macrophages. Parayath et al. utilized hyaluronic acid-poly(ethylenimine) polymer nanoparticles loaded with miR-125b for macrophage-targeted delivery and transfection in vivo.^[74]^ In peritoneal macrophages, miR-125b was increased by 100-fold leading to M1 macrophages repolarization for antitumor effects. In 2022, Chen et al. reported hydrogel-nanoporter complexes that creates glioma stem cells (GSCs) -specific CAR macrophages/microglia (CAR-Ms) in situ in the postsurgical cavity (Figure 3a).^[75]^ The hydrogel-nanoporter was synthesized using an amphipathic palmitoylated nuclear localization peptide, pCAR, dextran, brain extracellular matrix-derived peptide, and an immune-stimulating peptide. After intraluminal delivery, the hydrogel-nanoporter transferred CAR genes into widely distributed M2-type macrophages, leading to the generation of CAR-Ms in a glioma mouse model (Figures 3b,c). CAR-Ms exhibited M1-type macrophage features, which sought out and engulfed GSCs and cleared remaining GSCs in the tumor microenvironment (TME) by triggering adaptive anti-tumor immune responses. As illustrated in Figure 3d, the co-administration of the hydrogel-nanoporter and the aCD47 antibody led to an increase in positive immune cell response, resulting in improved survival rates and suppressed tumor growth.

Inorganic nanoparticles have been employed to reprogram macrophages in vivo. For instance, gold-based radiosensitive nano-regulator was used to generate reactive oxygen species and reprogram M2 macrophages into M1 macrophages in situ.^[76]^ In addition, Yang et al. reported silver nanoparticles coated with folic acid (FAAgNPs) for targeted delivery to M1 macrophages in the mouse model of rheumatoid arthritis (RA).^[77]^ The FAAgNPs showed anti-inflammatory activity in inflamed joints and improved the therapeutic efficacy in RA mouse models. This was attributed to the reduced number of M1-like macrophages through the triggered repolarization of macrophages. Furthermore, Kwon et al. synthesized mesoporous silica nanoparticles (XL-MSNs) for in vivo cytokine delivery to macrophages, which successfully induced M2-like macrophage polarization in vivo.^[78]^ In 2022, protoporphyrin IX (PpIX)-loaded manganese oxide (MnOx) with cell membranes acted as an in situ vaccine to activate macrophages from an M1-like phenotype to an M2-like phenotype for bacterial treatment in an osteomyelitis model.^[79]^

The polarization properties of macrophages provide advantages for in situ macrophage cell therapy. Polarized macrophages have the ability to rapidly and directly migrate to pathological sites, which benefits them by modulating the local microenvironment and accelerating the therapeutic effect while minimizing unnecessary cell toxicity and side effects.^[80,81]^ Even though in situ macrophage cell therapy is still in the preclinical stage, it shows promise as a potential treatment option for various diseases, including cancers, autoimmune diseases, and inflammatory disorders.^[81,82]^ However, the delivery efficiency of the therapeutic agent or nucleic acid molecule into macrophages is a key challenge. Macrophages are usually distributed in different tissues and organs, such as the liver, spleen, lung, and lymph nodes. Therefore, it is necessary to overcome the biological barriers of different tissues and cell types.

## 4 | DENDRITIC CELL BASED THERAPY

Dendritic cells (DCs) are a specialized type of antigen-presenting cells (APCs) that play a crucial role in initiating and regulating the adaptive immune response.^[83]^ DCs can activate cytotoxic T cells and helper T cells, which are important in combating infections and cancer.^[84]^ Therefore, DC therapy provides a powerful approach and pathway for combating tumors or other diseases.^[85]^ To date, one DC cell therapy product, Provenge^®^, has granted approval by the FDA for treating metastatic castrate-resistant prostate cancer.^[85]^ Because DCs derived from patients require activation ex vivo before injection into the body, challenges have severely hindered the widespread clinical utilization of DC-based cell therapy, including the poor homing efficiency, with less than 5% arriving at the draining lymph nodes,^[71]^ low response rates,^[86]^ high development costs and manufacturing.^[86–88]^ In situ activation of DC is the optimal protocol for maximizing DC cell therapy. Biomaterials with adjustable physicochemical properties and optimized release kinetics provide practical strategies to deliver antigens, adjuvants, or nucleic acid to DC cells in vivo.^[87,89]^

In the early 1990s, Martinon et al. utilized non-viral liposomes to deliver mRNA-encoding antigens into DCs to stimulate virus-specific cytotoxic T lymphocytes (CTLs) in vivo.^[90]^ In 2016, Katakowski et al. functionalized LNPs with an scFv that specifically targets murine DEC205 in vivo, a surface marker prominently expressed on CD8α^+^ DCs. These LNPs selectively targeted DEC205^+^ DCs in vivo.^[91]^ In LPS-activated mice, retro-orbital administration of LNPs knocked down nearly 70% of specific costimulatory targets (CD40, CD80, and CD86) in splenic DCs. More recently, Huang et al. developed cationic LNP for the delivery of siRNA targeting IDO1 (siIDO), which promoted DC maturation in tumor-draining lymph nodes in vivo. This LNP treatment showed strong antitumor immune responses for cancer immunotherapy.^[92]^ In 2020, Yu et al. developed a self-assembling melittin-LNP. These LNPs promoted the release of tumor-associated antigen and the activation of APCs, improving CD8^+^ T cells-mediated antitumor immunity.^[93]^ In 2022, Yan et al. formulated resiquimod (R848) derived amino lipids into RAL-LNPs, such as RAL1 and RAL2 LNP.^[94]^ Among them, RAL2-LNP delivered CD40 mRNA to DCs in vivo with high efficiency, facilitating DC maturation and inducing potent anti-tumor immune responses. Exosomes were another type of biomimetic material, which are similar to LNPs in that they are lipid bound particles but are extracellular vesicles derived from cells.^[95]^ Exosomes have also been used for in vivo DC cell therapy.^[96]^ In 2020, Elashiry et al. reported that DC-derived exosomes were encapsulated with TGFB1 and IL10, which increased DC maturation, recruited T-regulatory cells, and inhibited osteoclast-mediated degenerative bone loss.^[97]^

**FIGURE 4:**
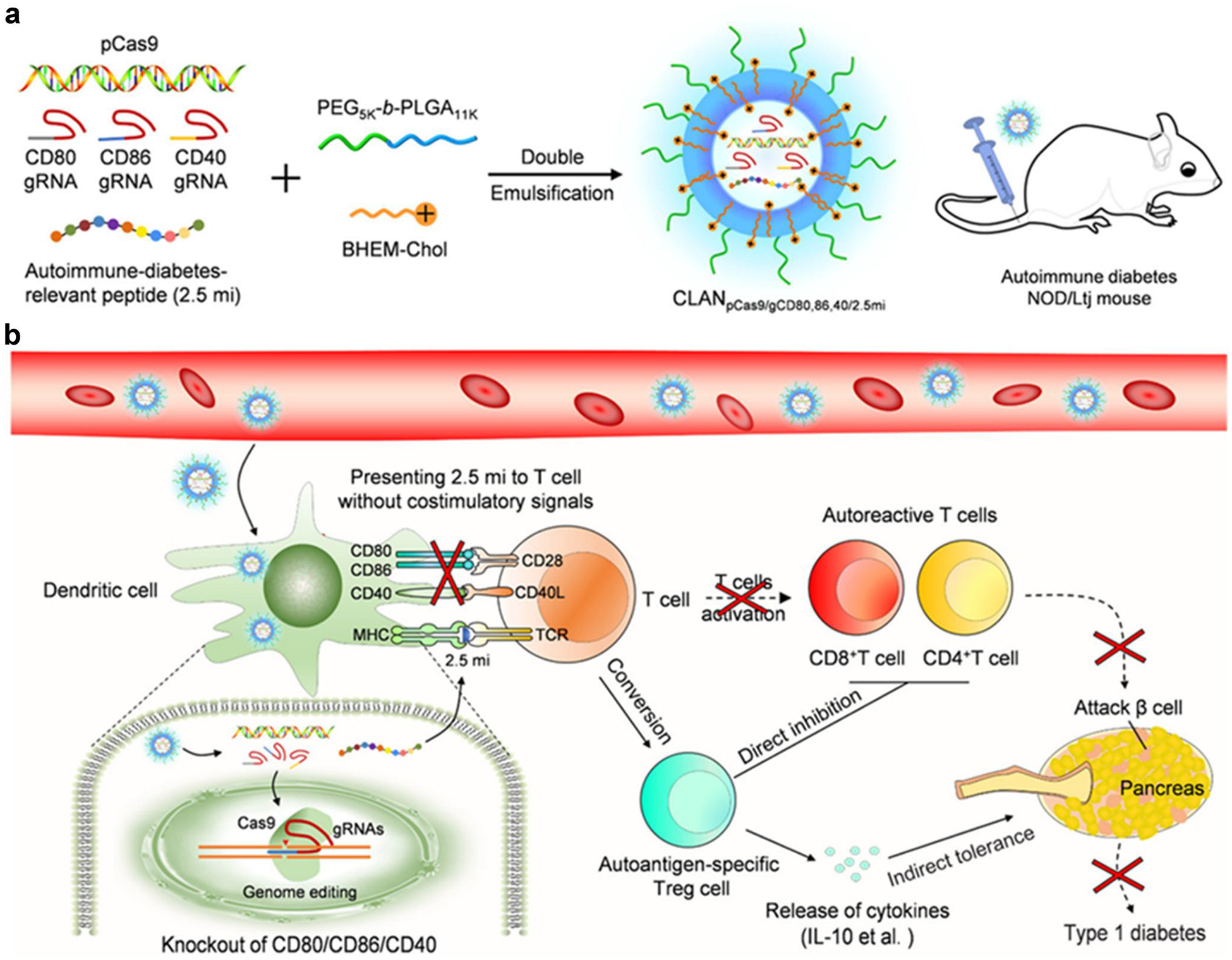
(a) Preparation of all-in-one nanomedicine, (b) Illustration of restoring specific immune tolerance (Reproduced with permission from ref 102.^[102]^ Copyright 2020 American Chemical Society).

Polymer-based biomaterials have also been explored for in vivo DC therapy.^[98]^ In 2014, Heo et al. developed PLGA nanoparticles to deliver poly I: C, STAT3 siRNA, and ovalbumin (OVA) antigen to DC. These polymer nanoparticles were able to overcome DC dysfunction and silence 50% STAT3 in tumor-associated DCs in vivo.^[99]^ Later, Zhou et al. designed a biomimetic nano-vaccine for improved cancer immunotherapy. The nano-vaccine consisted of a DMPC-PLGA core that was loaded with adjuvant imiquimod (R837). Then, the nano-vaccine was coated with an antigen peptide (αOVA) and apolipoprotein E3 (ApoE3) to enhance the uptake of antigens into DCs.^[100]^ In 2019, a chimeric cross-linked polymersome was reported to generate tumor-associated antigens, which promoted DC maturation in tumor-draining lymph nodes and inhibited tumor growth by a single injection.^[101]^ In 2020, Luo et al. used a polymeric complex based on poly (ethylene glycol)-*b*-poly(lactide-co-glycolide) (PEG-*b*-PLGA) to encapsulate a protein fragment (2.5 mi) relevant to autoimmune diabetes, a gene-editing plasmid (pCas9), and three targeting RNAs (aiming at CD40, CD80, and CD86) (Figure 4a,b).^[102]^ This multifunctional nanotherapeutic enabled DC targeting in a type 1 diabetic mouse model and engineering DC into a tolerogenic phenotype in situ. The 2.5 mi peptide was observed co-localization with MHC II^+^ DCs, while the pCas9/gCD80,86,40-mediated genome editing eliminated the co-stimulators CD80, CD86, and CD40 of DCs. As a result, the production of regulatory T cells (Tregs) was stimulated. Then, Treg cells released cytokines and protected pancreatic *β* cells to induce indirect tolerance for type 1 diabetes treatments. Using the same strategy, by encapsulating Cas9 mRNA and gCD40, the PEG-b-PLGA nanoparticles promoted the delivery into DCs and disrupted the costimulatory molecule’s expression on the DC surface, thereby impeding T-cell activation. In a mouse model of skin transplantation, these NPs decreased graft damage and extended graft survival.^[103]^ Meanwhile, Fan et al. reported a cationic LNP (CLAN) formulation, which was composed of PEG-PLGA and N, N-bis(2-hydroxyethyl)-N-methyl-N-(2-cholesterylox-ycarbonyl aminoethyl) ammonium bromide (BHEM-Chol). This formulation was utilized for the delivery of mRNA encoding OVA specifically to DCs.^[104]^ When tested in a lymphoma mouse model (E·G7-OVA), the treatment with CLAN produced a strong OVA-specific T-cell activation and inhibited tumor proliferation.

Inorganic biomaterials have emerged as potential modulators of DC immune responses in vivo.^[88]^ In 2010, Sokolova et al. showed that the activation of DCs can be achieved by calcium phosphate nanoparticles loaded with the TLR ligands CpG and poly(I:C).^[105]^ Since then, additional inorganic biomaterials such as gold nanoparticles and metal-organic frameworks have been explored to enhance DC maturation in vivo and improve cancer immunotherapy outcomes. In 2021, Xu et al reported that metal-organic frameworks constructed from manganese porphyrin delivered tumor-associated antigens to DCs in vivo, which increased mature DCs and reduced myeloid-derived suppressor cell amount.^[106]^

Consequently, in situ DC therapy can be targeted selectively to the site within the body, induce a specific immune response, present antigens to T cells, and potentially provide long-term therapeutic effects.^[107]^ Despite the enormous potential of DC therapy in vivo, the complexity and multiple mechanisms associated with the immune system may limit the development of this approach. The duration of biomaterial-induced immune tolerance has not been evaluated completely, and continuous responses are likely to require repeated administration. The long-term effects of tolerogenic biomaterials on safety and side effects also need to be studied.^[108]^ Furthermore, biomaterials possessing complicated components may encounter challenges in manufacturing for clinical use.

## 5 | STEM CELL BASED THERAPY

Stem cell-based therapy is an important therapeutic approach that primarily relies on the self-renewal and differentiation abilities of stem cells to treat diverse diseases.^[109,110]^ Stem cell therapy has been broadly adopted in clinical studies, culminating in FDA approvals for multiple stem cell therapy products based on hematopoietic and mesenchymal stem cells are approved by FDA currently.^[111–113]^ However, existing stem cell therapy requires ex vivo culture and differentiation followed by transplantation, which may present some challenges, such as difficulty in cell manipulation, low cell survival percentage, and high cost.^[114–116]^ Therefore, it is reasonable to consider in vivo therapy through stem cell differentiation in situ.^[117]^ The derivation of biomaterials to induce stem cell differentiation in situ is an emerging approach to stem cell therapy with numerous advantages.^[118]^ Biomaterials provide a biocompatible platform that enables the localization and differentiation of stem cells in vivo, which does not require the harvest of stem cells.^[119,120]^ Moreover, the use of biomaterials for stem-cell therapy in vivo has the potential to address challenges related to the efficacy and safety of ex vivo manufactured cells.^[121–123]^

**FIGURE 5:**
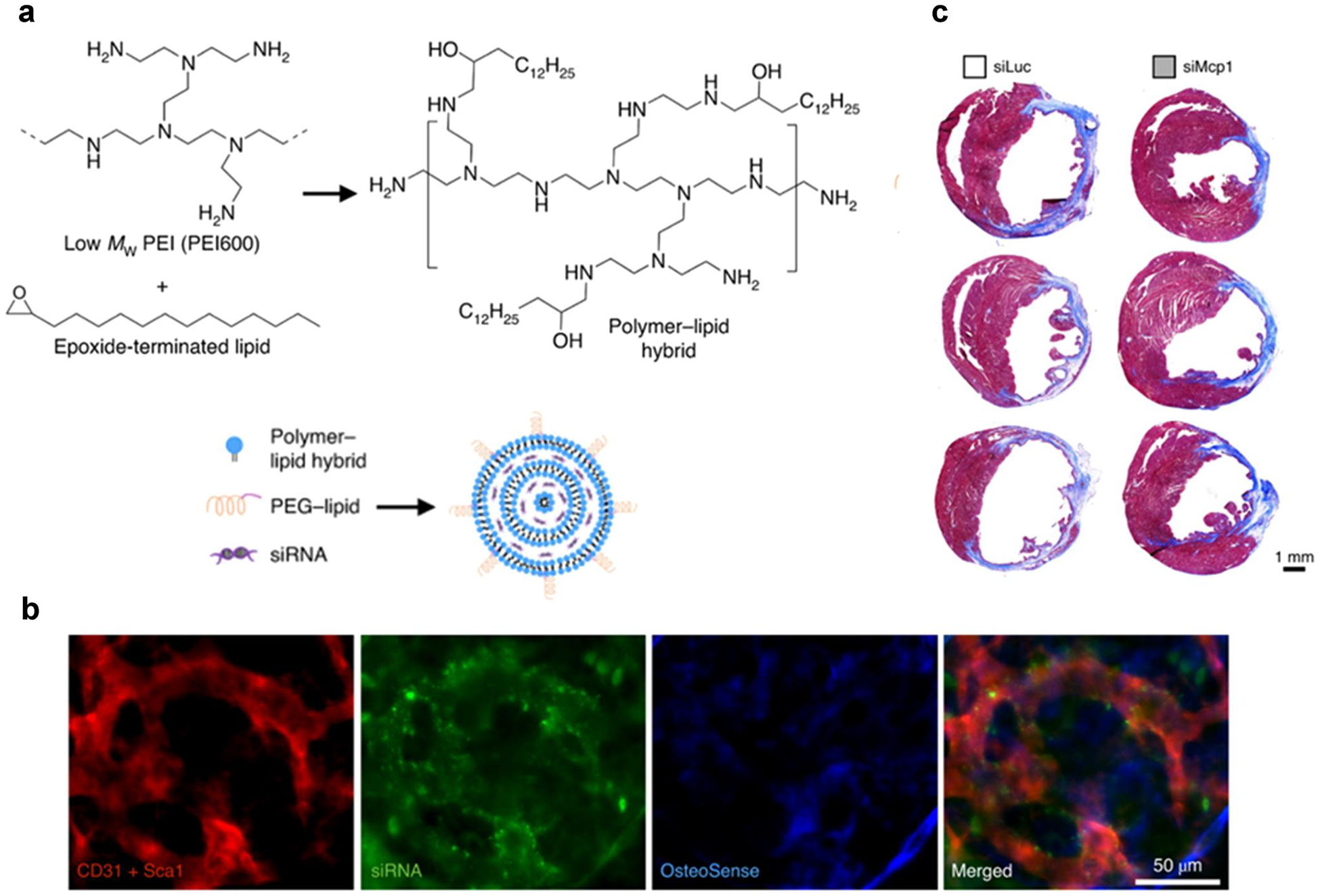
(a) Synthesis of lipid-polymer hybrid materials and nanoparticle formulation by microfluidic mixing, (b) After injecting NicheEC-15 with AF647-siRNA, colocalization was observed in the skull bone marrow within 2 h, (c) Masson’s trichrome staining of the left ventricle after NicheEC-15-siMcp1 treatment (Reproduced with permission from ref 130.^[130]^ Copyright 2020 Spinger-Nature).

Various biomaterials have been evaluated for in situ activation of stem cells. LNPs have been investigated for delivering RNA (miRNA or mRNA) to mesenchymal stem cells to induce neural-like differentiation.^[124,125]^ In early work, liposomes were synthesized and modified with apolipoprotein E (ApoE) to improve the delivery of siRNA or plasmid DNA to the brain.^[126,127]^ This strategy assisted the liposomes specifically targeting neural stem and progenitor cells. Shi et al. reported an active targeting strategy by evaluating a c-kit (CD117) antibody-modified LNP, which delivered RNA (siRNA and mRNA) to hematopoietic stem cell (HSC) in vivo. The results showed that administration of LNP-Cre recombinase mRNA enabled efficient gene editing in ~90% hematopoietic stem and progenitor cells (HSPCs) in mice. Furthermore, the edited cells maintain their stemness and functionality, leading to the production of mature immune cells.^[128]^ Synthetic polymers have also been studied for modulating stem cells in vivo.^[129]^ In 2020, Krohn-Grimberghe et al. explored lipid–polymer nanoparticles encapsulating siRNAs for gene silencing in the hematopoietic stem cell in vivo (Figure 5a).^[130]^ NicheEC-15, a lead formulation from the screening of epoxide modified lipid-polymer hybrid materials, exhibited effective delivery of siRNA to the hematopoietic niche (Figure 5b). After a single intravenous administration, NicheEC-15 nanoparticles loaded with angiopoietin-1 receptor (Tie2) siRNA induced approximately 80% suppression of Tie2 in vivo gene expression in the bone marrow. In a mouse model of myocardial infarction, NicheEC-15 nanoparticles encapsulated monocyte chemotactic protein 1 (Mcp1) siRNA, which reduced the presence of leukocytes in the diseased heart. Masson’s trichrome staining showed that a reduction in the scar area as a percentage of the left ventricle in mice treated with siMcp1, resulting in the restoration of cardiac function (Figure 5c). In 2022, El-Kharrag et al. utilized PBAE nanoparticles to deliver ribonucleoprotein to human granulocyte colony-stimulating factor (GCSF)-mobilized CD34^+^ hematopoietic stem cells. In comparison to electroporation, the utilization of PBAE nanoparticles for gene editing showed a 3-fold reduction in dosage requirement while maintaining similar efficacy. Additionally, these PBAE nanoparticles enhanced the engraftment capacity of human hematopoietic stem cells in the NSG mice xeno-graft model.^[131]^

Stem cells can differentiate into specific cell types to repair damaged tissue and regenerate functional cells.^[132,133]^ Thus, in situ stem cell therapy could be developed for a variety of therapeutic applications, such as cancers, Parkinson’s disease, diabetes, and spinal cord injury.^[134–136]^ However, it remains a tremendous challenge to utilize biomaterials for high-efficiency targeting and accessibility of hematopoietic stem cells or progenitor cells in vivo.

## 6 | CONCLUSION AND PERSPECTIVES

In situ cell therapy mediated by biomaterials has become a powerful strategy. This review summarizes recent advances in biomaterials to enable in vivo cell reprogramming, including T cells, macrophages, DCs, and stem cells. Compared to clinically approved cell therapy products, biomaterial-based in situ cell therapy can minimize complex procedures, reduce adverse effects, expand the patient population, and maximize therapeutic applicability. Therefore, in situ cell therapy based on biomaterials may provide broad clinical applications. However, several challenges need to be addressed before in situ cell therapy can be introduced into routine clinical practice.

The delivery efficiency in targeted cells and the safety of the biomaterial are considered major challenges for in situ cell therapy. Under complex physiological conditions, biomaterials encounter numerous obstacles during delivery in vivo, including physical and biological barriers. For example, biomaterials may be absorbed by proteins to form a protein corona and be rapidly cleared by the mononuclear phagocytic system or reticuloendothelial system, which can limit the delivery efficiency of biomaterial to target cells.^[137]^ Additionally, the delivery efficiency of biomaterial is affected by other aspects of the process for in situ cell therapy. On one hand, specific cell populations (e.g. T cell, macrophage, DC, and stem cell) may be present with different phenotypes and subtype functions in different organs.^[14]^ The delivery efficiency of biomaterials to specific cells could be reduced due to the lack of specific cell targeting ability. On the other hand, the interactions between biomaterials and specific cells can also affect the delivery efficiency. At present, most biomaterials depend on endocytosis for effective cellular uptake, but certain cell types, such as T cells, have non-phagocytic properties that reduce the delivery efficiency of biomaterial.^[13]^ Although the high dosage of biomaterials can improve drug delivery efficiency, they may also have potential side effects such as cytotoxicity and immune reactions. Therefore, one of the main research directions for the future of in situ cell delivery is the optimization and modification of biomaterial properties to improve delivery efficiency, reduce off-target accumulation and enhance dosage safety. This can be achieved in various manners. For example, PEG can be modified on the surface of biomaterials, thus increasing the circulation time of biomaterials.^[138]^ Also, ‘self’ peptides such as CD47 or cell membrane coatings can be used to extend circulation.^[139,140]^ For in situ cell therapy, unique characteristics of cell subsets need to be understood and identified thoroughly in the future to improve the targeting of biomaterials to cells with different phenotypes and subtypes. Reliable and well-defined biomarkers could be identified to effectively distinguish these cells in vivo. Based on these biomarkers, biomaterials can be designed to contain specific ligands, antibodies, peptides, or aptamers for precise targeting of specific cells in vivo.^[56,80]^ Furthermore, the design of biomaterials should take into account the influence of cellular uptake mechanisms. These mechanisms include but are not limited to phagocytosis, clathrin-mediated endocytosis, caveolin-mediated endocytosis, clathrin/caveolae-independent endocytosis, macropinocytosis, and receptor-mediated endocytosis.^[141–143]^ For example, cell-penetrating peptides or trans-membrane glycoprotein can be modified onto biomaterials to enhance cellular uptake efficiency, and biomaterials can also increase cellular uptake efficiency by interacting with the hydrophilic portion of the lipid bilayer.^[141,144]^ In addition, biomaterials may also be engineered with exogenous or endogenous responsiveness to allow controlled release of the cargo in target cells in vivo, which possibly improves safety and reduce off-target toxicity.^[145]^

Biomaterial-based in situ cell therapy is a fast-emerging field of cell therapy. This strategy has been applied to various therapeutic indications in preclinical animal models. Therefore, we believe that in situ cell therapy will be realized in the clinics with significant advances in biomaterials and understanding of cell therapy with acceleration.
